# Book Reviews

**Published:** 1822-02

**Authors:** 


### Monthly Medical and Scientific Bibliography.

Vexat censura . . . . . . Corvos.

### 1. GREAT BRITAIN.

Art. I.—*Introductory Lecture, delivered in the Theatre of the Royal College of Surgeons, on the* 8*th of May,* 1820. By B. C. Brodie, F.R.S. Professor of Anatomy and Surgery to the College, &c. 8vo. pp. 50. Burgess and Hill, London. 1820.

It is well observed by Mr. Brodie, that the dignity of our profession, and its rank in society, depend, in a great degree, on its scientific character; and that those who are anxious to uphold it in the estimation of others would do well to bear in mind, that, whenever its connexion with science is dissolved, it must sink to the level of meaner occupations. Of the truth of both these propositions, Mr. Brodie himself is,—and we trust will for many years to come, be a living example; no individual having, more earnestly than he has done, joined the pursuit of science with that of an arduous profession. The results have been such as ought to excite zeal and emulation among the junior classes of his brethren; for, while the profession has reaped great and lasting benefit from his exertions, public favour has called down upon him both honour and reward.

In his Introductory Lecture, it seems to be one of the principal objects of Mr. Brodie to impress on the minds of his audience the important maxim, "that the laws of life ought to be regarded as different from those which govern the changes of inorganic matter;" for, as the properties which are impressed on living beings are not only different from the properties of inorganic bodies, but possess, among themselves, very different characters, it follows that the phenomena presented by either must be different.

How infinitely more consoling to the mind of man must such a doctrine be, than that which would establish a pretended assimilation between matter at rest and ever-varying matter!

"One of the most remarkable circumstances belonging to life in its active state, is, that there is a perpetual change of the materials of which animate beings are composed. The old particles separate themselves in the form of the urine, the perspiration, and the various other excretions; and, to supply this waste, there is a constant influx of new particles from without. Dead inorganic matter becomes endowed with life, and forms a part of the substance of the living body, from which, after a certain period, becoming again detached, it returns to be blended once more with the external world, and to obey those laws to which it was originally subjected."

---

Art. II.—*An Essay on the Effects of the Fucus Helminthocorton upon Cancer, more especially in the Stage denominated Occult; with a concise Inquiry into the Origin and Nature of the Disease, &c.* By William Farr, m.c.s. &c. 8vo. pp. 112. W. Wright, London. 1821.

This little volume comes before the public with some claims to attention. It exposes many of the tricks of knavish quacks, as well as the errors of deluded and self-deceived practitioners; and contains the description of a novel method for treating one of the most formidable diseases to which mankind is subject. This method consists in the internal exhibition of a decoction, or infusion, of the *fucus helminthocorton*, or Corsican moss, once so celebrated as a vermifuge; and the form of the disease in which the medicine is stated to be of the greatest service, is that which has been termed *occult cancer*.

The facts adduced by the author in support of his practice, are few; but he is emboldened to say, that all the good effects which he has experienced in the dispersion of cancerous tumor will speedily be verified and confirmed by the experience and practice of others.

The author has indulged in some theoretic views, to account for the action of this medicine, and for the origin of the disease; but, for these he craves the indulgence of his readers.

Mr. Farr first gives a history of the method of cure; next, an account of the usual methods of treating cancer, with some severe animadversions on many of them. He then proceeds to enumerate the symptoms of cancer, remarking on some of them as he advances. His own method of treatment is then proposed, and detailed at full length. A few words occur on local treatment, which, in Mr. Farr's practice, consists chiefly of local warmth; and the conclusion follows.

Our own conclusion does not follow; because we are anxious to see, first, the report of other practitioners on the efficacy of this new remedy.

Mr. Farr is, of course, aware,—at least we imagine so,—that the fucus helminthocorton, being a marine plant, of the genus of those which furnish iodine, his proposition of administering it in occult cancers and glandular swellings, must be considered as a mere repetition of Dr. Coindet's proposal of using iodine in substance or in combination, both internally and externally, in the same class of diseases for which Mr. Farr recommends the Corsican moss. The question of administering iodine in glandular swelling engages, at this moment, the attention of the profession, both in this country and abroad; and we confess we were much surprised not to find the smallest notice taken of it in Mr. Farr's volume.

---

Art. III.—*A Medical Guide to the Cheltenham Waters: containing Observations on their Nature and Properties; the Diseases in which they are beneficial or hurtful; with the Rules to be observed during their Use.* By William Gibney, m.d. Graduate of the University of Edinburgh; Member of the Royal Medical Society of Edinburgh; one of the Physicians to the Cheltenham Dispensary, &c. 18mo. pp. xi. 162. Baldwin and Co. London.

We are quite puzzled as to the real character of this comely octodecimo. Does Dr. Gibney write for patients, or for *his* patients? To account for his coming forward with another volume on the subject of the Cheltenham waters, upon which so much has been lately published, the author asserts, that the greater number of those publications were written in a spirit of controversy; or are, by far, too extensive, and not adapted for books of reference. Under these impressions, he has undertaken the present work; and he trusts that it will prove practically useful.

This little volume is merely meant as a book of reference, and as an admonitory medical assistant to the patient; nothing being discussed which is not of practical importance. Dr. Gibney says, that, "a portable work of the kind has been for a long time a desideratum, and it is to be hoped the present essay may answer this intention."

The Introduction is purely geographical, statistical, and meteorological. The first part contains the "general character of mineral waters;" the second part treats of the "general effects of the Cheltenham waters." In the third part, we have a few words on the diseases in which the Cheltenham waters are useful; and these are—indigestion, hypochondriasis, stricture in the rectum, hepatic diseases, inflammatory disorders, rheumatism, ophthalmia, cutaneous affections, female complaints, and nephritic diseases. Those disorders where the Cheltenham waters are contra-indicated, are enumerated in a fourth part of the volume; and a fifth instructs us as to what should be done during a course of the Cheltenham waters,—the season, and the time of the day, for drinking them; the dose, the temperature, the duration of the course, and many other particulars, being equally noticed.

---

### 2. FRANCE.

Art. IV.—*Considérations sur une Altération Organique, appelée Dégénérescence Noire, Mélanose, Cancer Mélané, &c.* Par G. Breschet, Chef des Travaux Anatomiques a la Faculté de Médecine de Paris; Chirurgien en chef de l'Hospice des Enfans-Trouvés; Professeur d'Anatomie, de Physiologie, et de Chirurgie; Membre titulaire de l'Académie Royale de Médecine, &c. 8vo. pp. 24. Béchet, jeune. Paris, 1821.

The name, *melanoses*, was given, by Messrs. Bayle and Laennec, to certain small tumors, or collections of a dark yellow, bistre, or black matter, found within, or on the surface, of some animal tissues, which Professor Dupuytren was the first to notice. Dr. Breschet, who, in the course of his very numerous anatomical investigations, has had many opportunities of studying these morbid substances, conceives that they are the produce of secretion, rather than the result of decomposition, or disorganization of the tissue in which they are situated. He has found the black matter in question deposited in almost every organ of the animal economy; and has particularly observed it in the horse, as well as in other of the minor domestic animals.

The *melanic tumors*, of which Dr. Breschet has here undertaken to give a complete history, present themselves under various forms; all of which the author describes at full length, giving an account of their form and colour, as well as of their seat and texture. This affords him an opportunity for entering into several highly interesting and equally important anatomical details, many of which will be found perfectly new. It seems an indisputable fact, that these tumors are not organized. The author has seen some of them, through which there were nerves passing; but he could not perceive the smallest trace of any nervous filament being given out by the principal branch to the substance of the tumor.

The parenchyma of some of the viscera is very frequently found engorged with the melanic matter; particularly that of the lungs, liver, pancreas, and kidney. Laennec has mentioned several examples of melanosis of the lungs; so have Bayle, Esquirol, and Chomel, as well as Dr. Breschet himself, who has, also, had occasion to observe them in the mammary glands.

The author asks, "From what species of derangement of the animal economy can such melanic depositions arise? What are the changes that take place in the tissue in which the melanic matter is secreted? and by what distinguishing marks, general or local, can its existence be known during life?" He, at the same time, admits, that pathology and pathological anatomy have, as yet, supplied us with too scanty information to unveil these mysteries.

Patients, in whom the melanic tumors have been found after death, exhibited no particular symptoms of disease, indicative of that phenomenon, during life.

The chemical analysis of the black matter of melanic tumors confirms the idea of Dr. Breschet, that it is the produce of morbid secretion, and nothing else than decomposed blood. These tumors seem to be composed of coloured fibrine; of a black colouring matter soluble in diluted sulphuric acid and a solution of subcarbonate of soda, both of which are tinged red by its presence; of a small quantity of albumine; of chloruret of sodium, phosphate of lime, and oxide of iron.

It will easily be perceived, that this analysis assimilates the melanic tumors to coagulated blood. This is a highly-interesting subject, to which Dr. Breschet is very anxious to draw the attention of the anatomists in this country.

Art. V.—*Histoire des Mœurs et de l'Instinct des Animaux; avec les Distributions méthodiques et naturelles de toutes leurs Classes; cours fait à l'Athénée royal de Paris.* Par J. J. Virey, D.M. Professeur, &c. 8vo. 2 vols. With one volume of 182 Plates, representing 1100 Animals, forming an Atlas, in 8vo. Déterville, Paris.

Although it be one of the principal intentions of the Editors of this Journal to give an account of books on elementary or practical medicine, in preference to those of any other collateral science; it is by no means their wish to exclude from their pages the notice of any well-written or interesting work on the latter subject. It is, therefore, with pleasure that we announce the publication of a work, by the indefatigable Dr. Virey, which, whether we consider the matter or the composition, will be admitted, on all hands, to be both curious and interesting.

The first volume treats of the habits and manners of vertebrated animals,—namely, the mammiferous, birds, reptiles, and fishes. In the second, Dr. Virey describes the various and surprising instincts of the molluscæ, worms, crustacea, and insects; and, lastly, of zoophytes, including polypi, corals, and *animalculi infusorii*.

Dr. Virey contends that vertebrated animals possess, independently of instinct, different degrees of *acquired* intelligence; while the invertebrated animals are reduced to mere innate instinct, variously modified or developed, according to the family to which they belong. After each class of animals, the author presents the methodical and natural arrangement of the genera, agreeably to the most modern and exact notions in zoology. These volumes contain an animated picture of the numerous living beings scattered over the surface of the globe, each acting from the impulse of particular instincts, and whose attributes and habitations are accurately and pleasingly described.

The work may, in fact, be considered as a system of natural history of animals, drawn up in a manner calculated to edify as well as to instruct; and seems particularly intended to afford subjects for very interesting meditations.

---

### 3. ANSEATIC TOWNS.

Art. VI.—Influenza Europæa, *oder Diegrösserte krankeit Epidemie der neuern Zeit; or, General History of the most remarkable Epidemic Influenzas of modern Times.* By G. F. Most, M.D. 1 vol. 8vo. pp. xlviii. 254. Hamburgh.

The author first treats of the nature of *influenza*, and next enters into a consideration of its pathology, diagnosis, and mode of treatment. The second section contains a general history of epidemic influenzas, which he divides into three classes: the first embracing those influenzas which have prevailed, generally, throughout Europe, or in the greater part of it; the second, those which seem limited to particular countries; and the third, those which have been considered as epidemic influenzas, without sufficient reason.

The third section unfolds the occasional causes of influenza. The author observes, that it is an essential character of the disease to make its appearance in the north or north-east, and from thence to take a southerly direction. He endeavours to support his assertion by the citation of examples, where a cold easterly wind has produced severe catarrhs and catarrhal coughs; and, also, by a reference to experiments tending to show that those same diseases may be produced by the inspiration of an artificial gas.

The second part of this work contains a detail of the author's reasons for apprehending a fresh inroad of the Influenza Europæa; and lays down prophylactic rules for checking or impeding the progress of the epidemy which is to take place in the course of the current year, according to his observations. From these it appears, that the malady in question has shown itself every twenty years,—namely, in the years 1742, 1762, 1782, and 1802; although, at the commencement of the last century, the intervals were of a somewhat shorter duration. The author alludes to every recent meteorological phenomenon which comes in support of his prediction.

---

### 4. PRUSSIA.

Art. VII.—*Achter Bericht über die Merkwurdigern Krankheitsfalle, &c.; or, Eighth Report of the most remarkable Cases that have occurred in the Clinical Institution at Halle.* By Dr. Weinhold. 1821.

A man, forty-four years of age, of a weak constitution, had been afflicted, for upwards of eight years, with a considerable swelling of the scrotum, which reached as low down as the knees. This tumor was, in the first instance, mistaken for a sarcocele; and, subsequently, for a scrotal hernia. It was, at last, determined to make an incision into it; when a large quantity of feculent fluid issued out, and masses of hardened feces, weighing about three pounds, were removed. No communication, between the tumor and abdomen, could be discovered. Both testicles, as well as the deferential ducts, were hard, and forced on the outside of the swelling. At the upper part of the ring, a cribriform membrane was observed, through which liquid feces were constantly passing; and a swelling, formed by the abdominal integuments, over the right groin, appeared to contain a large portion of intestines and omentum. A considerable discharge of liquid matter from the wound took place, three days after the operation; and the patient recovered in ten weeks.

---

Art. VIII.—Jacobi Ludovici Conradi Schroeder van der Kolk, *Commentatio de Sanguinis vase Effluentis Coagulatione, &c.* 4to. pp. 61. Groningæ, 1820.

This memoir, on a prize-question, is full of interesting matter: it obtained the prize from the Academy of Groningen, and we think it fully deserved it. The author was restricted to the following points, in treating his subject;—1. To describe the phenomenon of coagulation of the blood, and to ascertain the particular state of the atmosphere most favourable to it. 2. To determine in what respects coagulated blood differs from that which is fluid. 3. To resolve the apparent contradictions existing in the results of Hewson, Hey, and Davy. 4. To explain the cause of coagulation, and the properties of the coagulum.

The phenomenon of coagulation is accurately and minutely described; and a curious microscopic description of the coagulum is given. The experiments of Hewson and Parmentier are alluded to; and five new ones, by the author, made to ascertain the influence of air on the phenomenon in question, are detailed at full length. The author states that, during coagulation, no heat is evolved; and that the blood, during that process, does not diminish in volume.

The conclusions which Dr. Schroeder thinks himself entitled to adopt from his inquiry, are the following:

"1. Coagulationis causa non extra corpus, sed iam in circulantis sanguinis fibrosâ parte invenitur.

"2. Hic nisus ab aëre et præcipue ab oxygenio oritur.

"3. Huic vero nisui ulterius perficiendo accedat vis vitalis, necesse est.

"4. Talis vis vitalis in sanguine adest, partique fibrosæ quendam irritabilitatis gradum conciliat.

"5. Coagulatio incepta instar fermenti cingenti sanguini communicatur.

"6. Nec calor corporis, nec motus circulatorius sanguinis, causæ sunt eius fluiditatis.

"7. In sanguine recenti motus, citissime rotantes, locum habent.

"8. Hi motus, vix tardiores, etiam in sanguine vasis contento, licet post mortem, se sustinere valent.

"9. In sero non tantum recenti, sed et vetustiori globulorum motus hic, licet tardiores, adsunt, reteque rubellum formatur.

"10. Illud itaque rete parti fibrosæ perverse adscribitur.

"11. Vires vitales, quæ motus gyrantes et rivulos producunt et sustinent, præcipuæ causæ videntur sanguinis fluiditatis."

---

### 5. HANOVER.

### Art. IX.—*Pharmacopœa Hannoveriana.* 8vo. pp. 40. Hanover, 1819.

Until the appearance of the present volume, there was no accredited collection of formulæ in the kingdom of Hanover; and the druggists, or apothecaries, prepared their medicines according to the Pharmacopœiæ which best suited their fancy or their convenience. The Prussian Pharmacopœia was the most in use. This great inconvenience has been, lately, remedied by the publication of the work before us. It is evident that the Berlin Pharmacopœia has served as a basis to the present one; but many additions and changes have been made to the former, and a great number of the preparations it contained have been omitted. Some of the processes, also, have been altered, and, we think, for the better.

The new nomenclature has been adopted; but a copious reference to the old denominations must render the change less inconvenient to those practitioners who have hitherto retained the latter.

Those articles which are of the first importance, and which ought always to be kept in a chemist's shop, are marked by an asterisk.

Some few remarks have been added on the virtues of every medicine; and the maximum dose of each noted, on the authority of Niemann.

In a short Appendix, we are informed of the *neue aizneitaxi für die koniglich*, &c. or new tax on drugs, for the kingdom of Hanover.

### 6. TUSCANY.

Art. X.—*Della Maniera più atta a Curare radicalmente le Varici ed Impiagamenti varicosi dell'Estremità inferiori, memoria di* Ranieri Cartoni. Small 8vo. pp. 93. Pisa, 1821.

The purport of this memoir is to prove, by a comparison of the various modes hitherto proposed for the treatment of varicose veins of the inferior extremities, and the ulcerations connected with them, as well as by new and recent cases, that the disease is best cured by the excision of the affected veins. Twenty cases are brought forward to prove that, where the ligature of the vein, only, has been affected, the advantage derived from it has not been, by any means, equal to that which is obtained by the cutting out of a portion of the diseased vessel.

### 7. SPAIN.

Art. XI.—*Noticia de una Niña que ha Nacido con Veinte y Cineo Dedos y Otras particularidades.* (Decadas Medico-Quirurgicas, tom. iv, No. 6.)

This is a remarkable case, transmitted by the resident physician of Valdeolivas, to the Editor of the Medical Journal mentioned in the above title. A girl was born, after a long and severe labour, having six fingers to each hand, as well as six toes to the left foot, while the right foot had seven. The child lived three days, but never opened its eyes. On separating forcibly the eye-lids, the cornea was observed to be covered by a thick yellow membrane, secreting an ichorous fluid.

### 8. UNITED STATES.

Art. XI.—*Case of a large Mass of an Amalgam of Tin and Mercury retained in the Bowels.* By J. Callup, m.d. (Philadelphia Journal, November 1821.)

The use of an amalgam of tin and mercury is not a novel subject to the profession: that is to say, the blue-pill, or calomel, has been given, on many occasions, in cases of worms, in combination with tin filings. In the cases related by Dr. Callup, an amalgam, previously made by uniting the two metals, was used thus:—A young man, thinking himself affected with worms, and having got a knowledge, from some almanack, that an amalgam of tin and mercury would destroy them, procured eight ounces of each of these articles; which, being made into an amalgam, were swallowed in doses of two ounces each, every two hours. No evacuation of either worms or the amalgam followed the ingestion of the metals; and the patient lived four years and a half, without apparently feeling any inconvenience from the metallic ball, which, according to Dr. Callup, who had examined the patient, could not weigh less than a pound. The man died at St. Charles, in Missouri, of the fever of the climate; his health having considerably improved before that time; and never having complained of worms from the moment he swallowed the amalgam.

